# Wildfire smoke exposure and persistent respiratory symptoms and illness: a retrospective cohort study

**DOI:** 10.1186/s12931-026-03694-3

**Published:** 2026-05-12

**Authors:** Gabrielle Y. Liu, Sean Raffuse, Mira Miles, Mariela Alaniz, Lusine Gigoyan, Nicholas J. Kenyon, Ravi Kalhan, Irva Hertz-Picciotto

**Affiliations:** 1 Division of Pulmonary, Critical Care, and Sleep Medicine, Department of Internal Medicine, University of California Davis, Sacramento, USA; 2 Air Quality Research Center, University of California Davis, Davis, USA; 3 Department of Public Health Sciences, University of California Davis, Davis, USA; 4 Division of Pulmonary and Critical Care Medicine, Department of Medicine, Northwestern University Feinberg School of Medicine, Chicago, USA

**Keywords:** Air pollution, Environmental health, Wildfire smoke, PM2.5, Epidemiology, Population health, Pulmonary medicine, Asthma

## Abstract

**Background:**

Wildfire smoke is a growing public health hazard yet there is limited evidence on its intermediate or long-term respiratory health effects. Studying the association between wildfire smoke exposure and indicators of impaired respiratory health such as persistent respiratory illness and respiratory symptoms can help elucidate the associated risk of developing chronic lung disease. We sought to determine whether wildfire smoke exposure is associated with medically-attended respiratory illness (MARI) and respiratory symptoms persisting beyond the acute wildfire period.

**Methods:**

This was a population-based retrospective cohort study of adults without chronic lung disease aside from self-reported asthma, who reported living in a Northern California community affected by a wildfire in 2018. The primary predictor was mean daily wildfire-dominated fine particulate matter (PM_2.5_) exposure during a wildfire, estimated based on home and evacuation locations. The primary outcomes were respiratory illness requiring medical attention (MARI) at least one month after the wildfire and any respiratory symptom (“asthma attack,” bronchitis, cough, respiratory infection, or wheezing) that persisted to the time of the survey (mean 8.5 months after wildfire).

**Results:**

Among 1,381 adults in the study, the mean daily PM_2.5_ exposure during a wildfire was 87.2 μg/m^3^ (SD 44.3). After adjustment for demographic covariates, smoking status, asthma, and allergies, one standard deviation higher wildfire-dominated PM2.5 exposure was associated with greater risk of MARI (RR 1.18, 95% CI 1.06 - 1.31, *p*=0.002) and persistent self-reported asthma attacks (RR 1.28, 95% CI 1.03 - 1.59, *p*=0.026), but not associated with other persistent respiratory symptoms. Each additional day exposed to PM_2.5_ >125 μg/m^3^ (EPA threshold for very unhealthy or hazardous air quality) was associated with an 11% increased risk of future severe MARI leading to emergency room visit or hospitalization (RR 1.11, 95% CI 1.02 – 1. 20, *p*=0.018).

**Conclusions:**

Exposure to wildfire-dominated PM_2.5_ is associated with increased risk of respiratory illness and symptoms which persist for months beyond the acute wildfire period and may indicate risk of future chronic lung disease. Additional days of exposure to very unhealthy or hazardous range PM_2.5_ levels are associated with increased risk of ongoing respiratory morbidity and emergency healthcare utilization in the adult general population.

**Supplementary Information:**

The online version contains supplementary material available at 10.1186/s12931-026-03694-3.

## Introduction

Exposure to smoke from wildfires is a growing public health threat. As the earth’s temperature rises, the size and severity of wildfires have increased over time [1–3]. The link between wildfire smoke and short-term respiratory morbidity is clear, as evidenced by well-documented increases in emergency room (ER) visits, hospital admissions, and respiratory deaths associated with wildfire smoke exposure [4–6]. However, the intermediate and long-term respiratory health effects of exposure to wildfire smoke have received far less attention and are challenging to study given the need for prolonged follow-up time to detect incident chronic lung disease.

Impaired respiratory health has been defined as an intermediate phenotype on the continuum between ideal lung health and chronic lung disease [7]. As such, indicators of impaired respiratory health can help identify persons at risk of chronic lung disease. Persistent respiratory symptoms reported by adults without lung disease are one such indicator and have been associated with incident lung function impairment and emphysema [8, 9]. Additionally, it is well-documented that acute respiratory exacerbations and respiratory illness requiring medical attention (medically-attended respiratory illness [MARI]) occur among those with and without chronic lung disease, are a major driver of respiratory-related morbidity, and are associated with incident accelerated decline in lung function and increased mortality [10–14]. 

We hypothesized that wildfire smoke exposure is associated with MARI and respiratory symptoms that persist beyond the acute wildfire period, informing risk of future chronic lung disease. We investigated this hypothesis by analyzing data from a survey of adults without chronic lung disease who were living in Northern California during the July-November 2018 wildfire season. Most participants in this survey reported being affected by the 2018 Camp Fire, a large wildland-urban interface (WUI) wildfire which caused 85 deaths, destroyed 18,804 structures, and remains the deadliest and most destructive wildfire in California history [15]. 

## Methods

### Study design and population

This is a retrospective cohort study using data from the ‘***W***ildfires and ***H***ealth: ***A***ssessing the ***T***oll in ***No***rthern ***Ca***lifornia Study’ (WHAT-Now, CA) Study [16]. In May 2019, the online WHAT-Now survey was made available in English and Spanish with a focus on participant experiences during and after the 2018 Northern California wildfires. The present analysis included any adult respondent without pre-existing lung disease other than asthma living in Northern California who reported being primarily affected by a 2018 wildfire. This study was performed in accordance with the Declaration of Helsinki and was approved by the University of California, Davis Institutional Review Board before any recruitment or data collection. All respondents provided informed consent to participate. Detailed methods are presented in the Supplement.

### Wildfire-dominated PM_2.5_

In the baseline survey, participants indicated which 2018 Northern California wildfire affected their community and gave the address of where they were living at the onset of that wildfire. Participants who evacuated also recorded what county (or counties) they evacuated to, the date of arrival to each location, whether they returned home, and the date they returned home. In a follow-up survey, participants who evacuated were asked to give the address(es) of the location(s) they evacuated to.

**Fig. 1:**
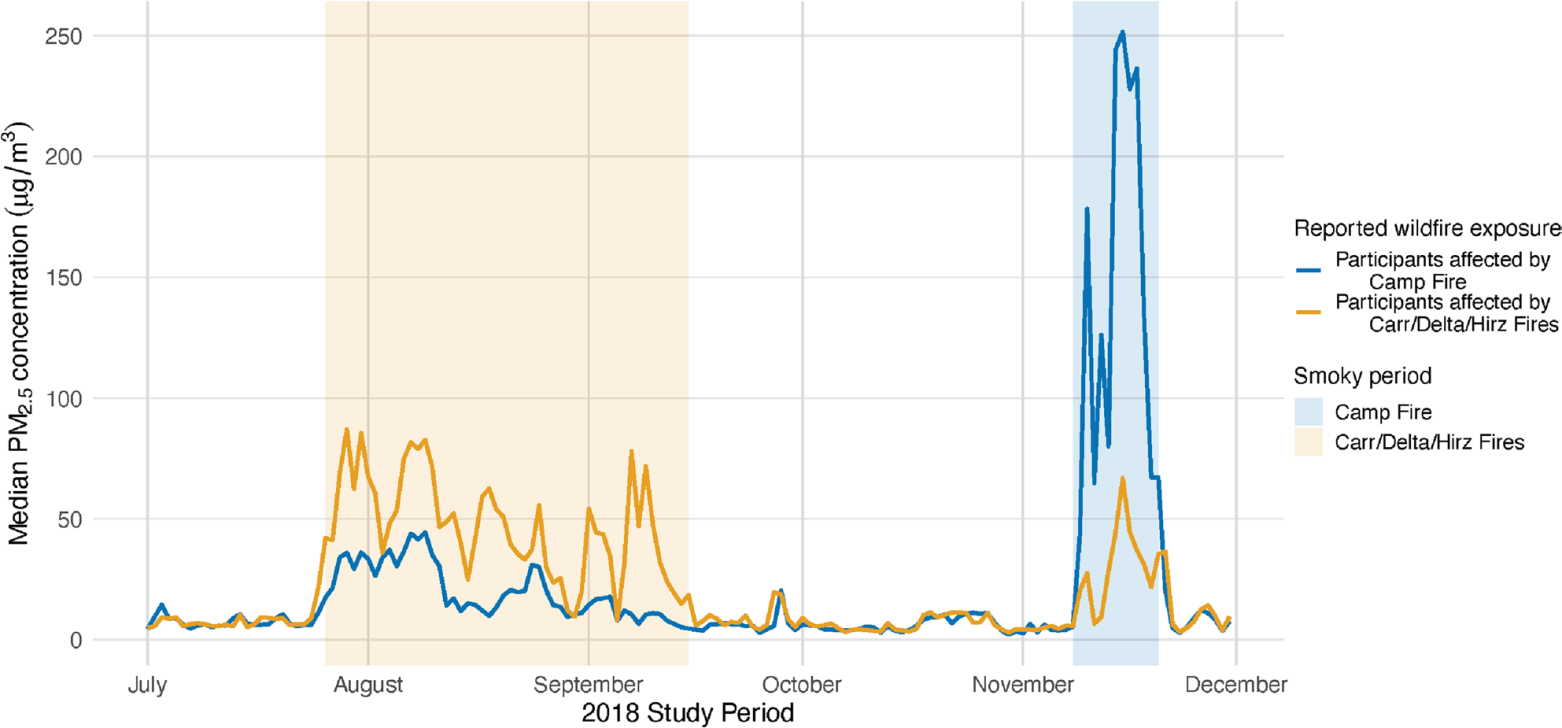
Daily median PM_2.5_concentrations among participants by reported wildfire exposure. Based on home locations of participants who reported being affected by the Camp, Carr, Delta, or Hirz wildfires (95% of the overall cohort). Smoky periods for each wildfire were determined to start on the first day of the wildfire and end when the mean daily PM_2.5_ concentration for the affected area returned to prior baseline levels. Participants who reported being affected by the Carr, Delta, or Hirz fires had the smoky periods from all three fires combined as these fire periods were overlapping and occurred in the same counties (Shasta County, Trinity County). The smoky period for the Carr, Delta, Hirz Fires was 7/26/2018 to 9/15/2018 and the smoky period for the Camp Fire was 11/8/2018 to 11/20/2018

Using participant home address, or evacuation address(es) when available, 24-hour mean surface concentrations of fine particulate matter (PM_2.5_) were estimated for each day in the study period (July 1 to November 30, 2018, which encapsulates the period of major Northern California wildfires in 2018). Detailed methods on the PM_2.5_ model have been previously described and it was demonstrated to have high correlation with PM_2.5_ levels at permanent monitoring sites [17]. In brief, daily 24-hour concentrations of total PM_2.5_ were estimated using a random forest model with data from sources particularly important during wildfire events: a regional air quality model, ground-based monitoring, low-cost sensors, satellite-derived aerosol optical depth, and meteorological parameters. The primary exposure was the mean daily PM_2.5_ concentration during the wildfire-specific smoky period (“wildfire-dominated PM_2.5_”), calculated using participants’ daily locations. Smoky periods for each wildfire are shown in Table S1 and were determined to start on the first day of the wildfire and end when the median daily PM_2.5_ concentration for the affected area returned to prior baseline levels (Fig. 1). Total PM_2.5_ concentration during these periods is termed “wildfire-dominated PM_2.5_” given that PM_2.5_ levels during these wildfires are substantially higher than typical PM_2.5_ levels in these areas due to wildfire smoke (Fig. 1). When participants reported evacuating but did not specify an evacuation address, their daily 24-hour PM_2.5_ levels were imputed based on the mean 24-hour PM_2.5_ concentration of the county(ies) they evacuated to and the reported days they were in each county.

In secondary analyses, we examined the risk of MARI and severe MARI associated with additional PM_2.5_ metrics during the person-specific exposure period: peak PM_2.5_, peak week mean PM_2.5_ (daily average over the 7 consecutive days with highest PM_2.5_ concentrations), total mean PM_2.5_ (daily average over entire exposure period), and number of days during the exposure period with PM_2.5_ greater than 35 µg/m^3^ (U.S. Environmental Protection Agency (EPA) 24-hour PM_2.5_ standard), 55 µg/m^3^ (EPA threshold for unhealthy air quality) and 125 µg/m^3^ (EPA threshold for very unhealthy air quality). The exposure period was July 1 to November 30, 2018, with exposure censored at the first MARI or severe MARI event to ensure exposure preceded outcome. Because the exact timing of MARI events was not available, event timing was approximated using the earliest positive follow-up timepoint (1, 3, or 6 months after the start date of the wildfire reported by the participant). For example, if a participant reported MARI at 1 month after the wildfire, their exposure period would end 1 month after the start of the wildfire they were exposed to.

### Outcomes

The primary outcomes for this study were self-report of MARI and any persistent respiratory symptom. Participants were asked in the baseline survey whether they had asthma attack, bronchitis, cough, respiratory infection, or wheezing or whistling in the chest, at each of the following time points after the wildfire: first 3 weeks, 1 month, 3 months, 6 months after, and currently, i.e., at the time the survey was taken (between 6.2 and 19.6 months post-fire). A respiratory symptom was considered “persistent” if they endorsed having the same symptom “currently” and at least one other post-wildfire timepoint. To examine respiratory illness after the acute wildfire period, MARI was defined by self-report of seeing a doctor or nurse, visiting the ER or being hospitalized for their respiratory symptom *at least one month after* the start of the wildfire. Severe MARI was defined as visiting the ER or being hospitalized for the respiratory symptom.

### Statistical analysis

Multivariable Poisson regression with robust standard error was used for all analyses. Models were complete-case analysis and included age, sex, self-identified race/ethnicity, active tobacco smoking, education, pre-existing asthma, and pre-existing environmental allergies as covariates. For the secondary analyses examining the association between MARI or severe MARI and PM_2.5_ metrics during the person-specific exposure period, these models were additionally adjusted for the duration of an individual’s exposure window to account for differences in exposure opportunity across participants. Exploratory subgroup analyses examined whether certain demographic characteristics or personal smoke mitigation methods modified the association between wildfire-dominated PM_2.5_ and the outcomes of interest. Given that these were exploratory analyses, we used a p-value threshold of 0.20 and did not adjust for multiple testing. Analyses were performed using R software (version 4.2.3) and RStudio (version 2024.04.2 + 764).

## Results

**Fig. 2:**
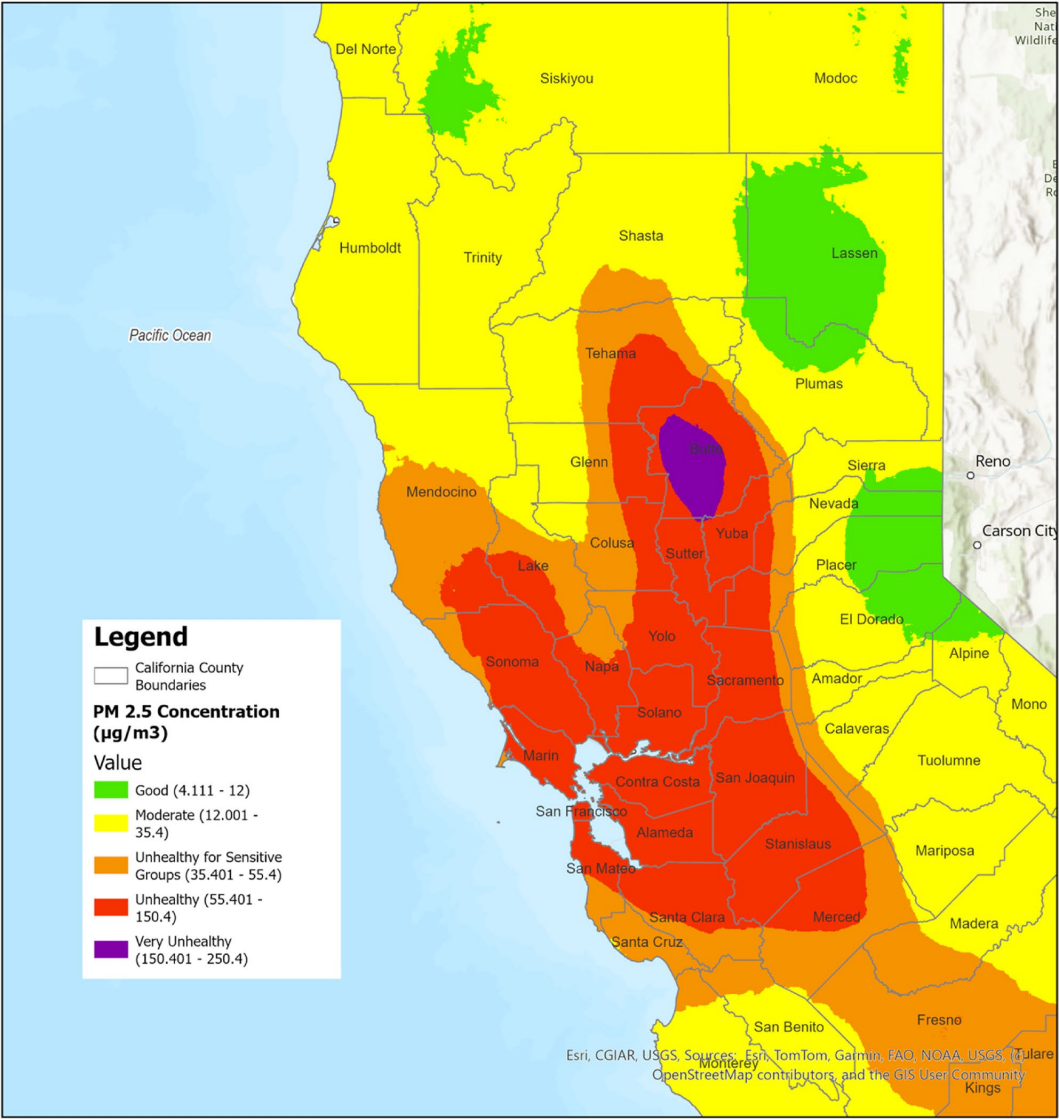
Mean daily wildfire-dominated PM_2.5_ level during the 13 smoky days of 2018 Camp Fire, by Northern California county. Daily 24-hour concentrations of PM_2.5_ were estimated using a random forest model with data from sources particularly important during wildfire events: a regional air quality model, ground-based monitoring, low-cost sensors, satellite-derived aerosol optical depth, and meteorological parameters. The California counties where the highest proportions of participants resided were Butte (56%), Shasta (15%), Sacramento (5%), Siskiyou (4%)

This study included 1,381 adult respondents who did not report a pre-existing history of lung disease other than asthma. Roughly half of the participants (702, 51%) evacuated during the wildfire period and of those participants, 694 (99%) reported both evacuation location(s) and date(s) of arrival. Among those who evacuated, 328 (47%) had not returned home by the time of the survey. Of the 327 participants who reported returned home, 325 (99.4%) reported a return home date. These locations and dates were used to estimate daily 24-hour concentrations of PM_2.5_ during the study period. The overall cohort mean wildfire-dominated PM_2.5_ exposure during smoky days based on participant home or evacuation location(s) was 87.2 µg/m^3^ (SD 44.3). There were high proportions of respondents identifying as female, White, or as non-Hispanic, and this did not significantly differ by level of wildfire-dominated PM_2.5_ exposure (Table 1). Rates of evacuation were lowest among participants with the highest wildfire-dominated PM_2.5_ exposure. There were 1,051 respondents (76%) who reported being primarily affected by the 2018 Camp Fire (Table S1). Figure 2 displays the mean wildfire-dominated PM_2.5_ level during the 13 smoky days of the 2018 Camp Fire by region in Northern California. Table 2 displays the cohort-level mean, standard deviation, median, and interquartile range for each PM_2.5_ metric.

**Table 1:** Baseline characteristics by wildfire-dominated PM2.5 exposure during smoky period

Characteristic	Low PM_2.5_ *N* = 276	Medium PM_2.5_ *N* = 801	High PM_2.5_ *N* = 304
Mean PM_2.5_ level on smoky days in µg/m^3^	28.59 (9.90)	84.15 (26.88)	148.33 (4.94)
Age: mean (SD)	50.61 (14.27)	49.58 (15.35)	49.28 (14.92)
Female	236 (86%)	667 (83%)	257 (85%)
Race			
Multiracial	23 (8.8%)	56 (7.5%)	8 (2.8%)
*Other	7 (2.7%)	25 (3.4%)	8 (2.8%)
White	230 (88%)	661 (89%)	266 (94%)
Ethnicity			
Hispanic or Latino/a	20 (7.7%)	51 (7.0%)	13 (4.6%)
Not Hispanic or Latino/a	240 (92%)	681 (93%)	270 (95%)
Education			
Graduate or professional degree	48 (19%)	154 (21%)	56 (20%)
Bachelor’s degree	76 29%)	183 (24%)	88 (31%)
Some college (no degree)	67 (26%)	209 (28%)	66 (23%)
Vocational, Technical, or Associate degree	47 (18%)	141 (19%)	55 (19%)
High school (or GED) or less	21 (8.1%)	62 (8.3%)	20 (7.0%)
Current smoker	39 (14%)	124 (16%)	45 (15%)
Prior asthma	71 (26%)	191 (24%)	63 (21%)
Prior allergies	109 (39%)	337 (42%)	136 (45%)
Evacuated	145 (53%)	457 (57%)	100 (33%)
Used indoor air purifier during fire	88 (32%)	264 (33%)	101 (33%)
Kept windows closed	249 (91%)	712 (90%)	291 (96%)
Outdoor activities during fire	94 (34%)	210 (26%)	79 (26%)
Wore N95 mask outdoors			
No	127 (47%)	286 (36%)	34 (11%)
Yes, a lot	67 (25%)	282 (36%)	160 (53%)
Yes, sometimes	78 (29%)	226 (28%)	108 (36%)

Low, medium, and high PM_2.5_ group were defined as > 1 SD below the mean, within ± 1 SD of the mean, and > 1 SD above the mean, respectively

Percentages calculated among participants with non-missing data. Missingness in the low PM_2.5_ group ranged from 0% (age, prior asthma, prior allergies, evacuated, outdoor activities during fire) to 6.1% (education); among the medium PM_2.5_ group, 0% (age, sex, prior asthma, prior allergies, evacuated) to 8.6% (ethnicity); and among the high PM_2.5_ group, 0% (age, sex, prior asthma, prior allergies, evacuated, kept windows closed, outdoor activities during fire) to 7.2% (race)

PM_2.5_ Fine particulate matter, *SD *standard deviation, *GED *general educational development test, equivalent to high school degree in the United States

*Asian, Pacific Islander, Black, Native American, Alaska Native, or Other race categories combined to suppress cells with less than 5 participants and preserve confidentiality

**Table 2:** Summary of cohort-level PM_2.5_ exposure measures during study period (July to November 2018)

PM_2.5_ measure	Mean	SD	Median	IQR
Mean daily PM_2.5_ over smoky days (µg/m^3^)	87.2	44.3	86.7	75.1
Peak PM_2.5_ (µg/m^3^)	205.3	94.2	233.5	168
Peak week mean PM_2.5_ (µg/m^3^)	131.9	62.1	124.9	109
Total mean PM_2.5_ (µg/m^3^)	20.6	5.8	20.3	7
Days with PM_2.5_ > 35 µg/m^3^	21.0	9.0	20	11
Days with PM_2.5_ > 55 µg/m^3^	10.4	4.5	10	3
Days with PM_2.5_ >125 µg/m^3^	3.6	3.2	3	6

Mean daily PM_2.5_ over smoky days (wildfire-dominated PM_2.5_) was calculated as the average of the daily 24-hour PM_2.5_ levels from the smoky days of the wildfire that participants reported being primarily affected by. Additional PM_2.5_ exposure metrics were calculated over the person-specific exposure period (July 1–November 30, 2018), with exposure censored at the first MARI event. These included: peak PM_2.5_ (maximum 24-hour PM_2.5_ level during the exposure period); peak week mean PM_2.5_ (average 24-hour PM_2.5_ level over the 7 consecutive days with the highest concentrations); total mean PM_2.5_ (mean 24-hour PM_2.5_ level over the entire exposure period); and the number of days with PM_2.5_ >35, 55, 125 µg/m^3^, corresponding to the U.S. EPA’s 24-hour PM_2.5_ standard, threshold for unhealthy air quality (AQI > 150), and threshold for very unhealthy air quality (AQI > 200), respectively

*SD *standard deviation, *IQR *interquartile range

### Medically-attended respiratory illness

The overall prevalence of having any MARI at least one month after the start of the wildfire was 21.9% and the prevalence of having any severe MARI leading to ER visit or hospitalization was 4.1% (Table 3). In fully adjusted models, wildfire-dominated PM_2.5_ exposure during smoky days was associated with an increased risk of MARI (RR 1.18 per SD [95% CI 1.06–1.31] or RR 1.04 per 10 µg/m^3^ [1.01–1.06]). MARI was also significantly associated with total mean daily PM_2.5_ level over the entire person-specific exposure period (RR 1.19 per 10 µg/m^3^ [1.05–1.34]) (Table 4). Severe MARI was associated with peak PM_2.5_ (RR 1.03 per 10 µg/m^3^ [1.001–1.06]) and number of days with PM_2.5_ level in the very unhealthy to hazardous range (> 125 µg/m^3^) (RR 1.11 per additional day, [1.02–1.20]).

**Table 3:** Wildfire-dominated PM2.5 exposure during smoky period and respiratory outcomes after the acute wildfire period

Outcome	Prevalence	RR per SD (95% CI)	RR per 10 µg/m^3^ (95% CI)	*p*-value
Any episode of MARI	302/1381 (21.9%)	**1.18 (1.06–1.31)**	**1.04 (1.01–1.06)**	**0.002**
Any episode of severe MARI	56/1381 (4.1%)	1.28 (0.99–1.66)	1.06 (1.00–1.12)	0.061
Persistent cough	139/1381 (10.1%)	1.16 (0.99–1.37)	1.03 (1.00–1.07)	0.067
Persistent asthma attacks	60/1381 (4.3%)	**1.28 (1.03–1.59)**	**1.06 (1.01–1.11)**	**0.026**
Persistent bronchitis	24/1381 (1.7%)	1.18 (0.77–1.81)	1.04 (0.94–1.14)	0.446
Persistent wheezing	68/1381 (4.9%)	1.1 (0.85–1.42)	1.02 (0.97–1.08)	0.452
Persistent respiratory infections	16/1381 (1.2%)	1.22 (0.78–1.91)	1.04 (0.95–1.15)	0.393
Any persistent respiratory symptom	189/1381 (13.7%)	1.13 (0.99–1.3)	1.03 (1.00–1.06)	0.076

The mean wildfire-dominated PM_2.5_ exposure was calculated as the average of the daily 24-hour PM_2.5_ levels from the smoky days of the wildfire that a participant reported being primarily affected by. Results from multivariable Poisson regression models with robust standard error using continuous PM_2.5_ z-scores (SD) or using per 10 µg/m^3^ increase, adjusted for age, sex, race/ethnicity (Non-Hispanic White vs. all other race/ethnicity groups), current smoking status, pre-existing asthma, pre-existing environmental allergies, education (Bachelor’s degree or higher vs. less than Bachelor’s degree)

*RR *relative risk, *MARI *medically-attended respiratory illness at least 1 month after the wildfire, *Severe MARI*  respiratory illness leading to emergency room visit or hospitalization

*P*-value < 0.05 bolded

**Table 4:** PM_2.5_ metrics and respiratory symptoms after acute wildfire period

Outcome	Peak PM_2.5_	Peak week mean PM_2.5_	Total mean PM_2.5_	Days with PM_2.5_ >35 µg/m^3^	Days with PM_2.5_ >55 µg/m^3^	Days with PM_2.5_ >125 µg/m^3^
	RR per 10 µg/m^3^ (95% CI)			RR per additional day (95% CI)		
Any episode of MARI	1.01 (1.00, 1.02)	1.01 (1.00, 1.03)	**1.19** **(1.05**,** 1.34)**	0.99 (0.98, 1.00)	0.99 (0.96, 1.01)	1.03 (0.99, 1.06)
Any episode of severe MARI	**1.03** **(1.001**,** 1.06)**	1.04 (1.00, 1.08)	0.94 (0.61, 1.47)	1.00 (0.97, 1.02)	0.99 (0.94, 1.04)	**1.11** **(1.02**,** 1.20)**

PM_2.5_ exposure metrics were calculated over the person-specific exposure period (July 1–November 30, 2018), with exposure censored at the first MARI event. These included: peak PM_2.5_ (maximum 24-hour PM_2.5_ level during the exposure period); peak week mean PM_2.5_ (average 24-hour PM_2.5_ level over the 7 consecutive days with the highest concentrations); total mean PM_2.5_ (mean 24-hour PM_2.5_ level over the entire exposure period); and the number of days with PM_2.5_ >35, 55, 125 µg/m^3^, corresponding to the U.S. EPA’s 24-hour PM_2.5_ standard, threshold for unhealthy air quality (AQI > 150), and threshold for very unhealthy air quality (AQI > 200), respectively. Results from multivariable Poisson regression models with robust standard error adjusted for age, sex, race/ethnicity (Non-Hispanic White vs. all other race/ethnicity groups), current smoking status, pre-existing asthma, pre-existing environmental allergies, education (Bachelor’s degree or higher vs. less than Bachelor’s degree)

*RR *relative risk, *MARI *medically-attended respiratory illness at least 1 month after wildfire, *Severe MARI*  respiratory illness leading to emergency room visit or hospitalization, *EPA* Environmental Protection Agency

*P*-value < 0.05 bolded

### Persistent respiratory symptoms

**Fig. 3:**
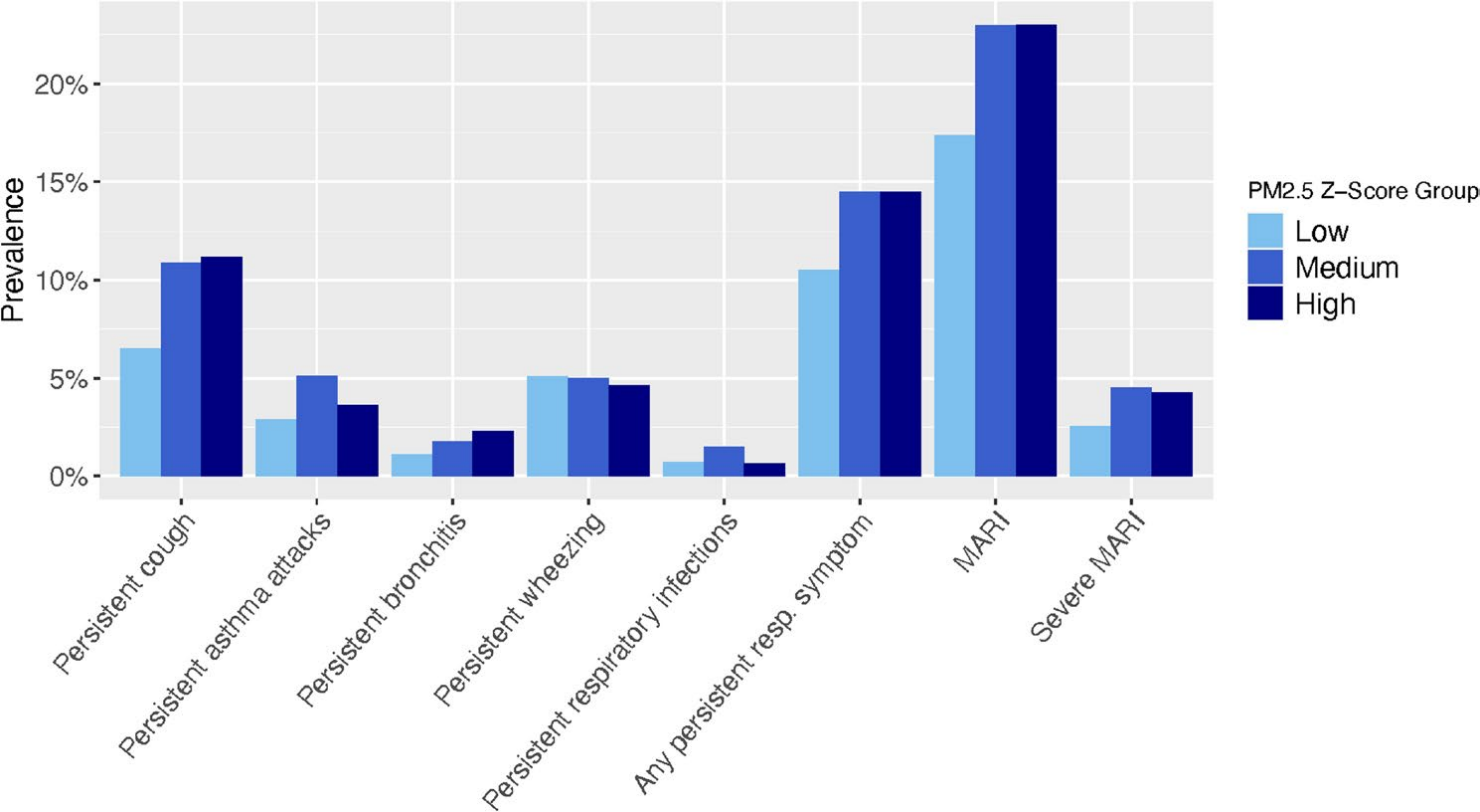
Prevalence of persistent respiratory symptoms, medically-attended respiratory illness (MARI), and severe MARI, by low, medium and high wildfire-dominated PM_2.5_ exposure during smoky period. Low, medium, and high PM_2.5_ group were defined as > 1 SD below the mean, within ± 1 SD of the mean, and > 1 SD above the mean, respectively. MARI was defined by participant report of respiratory symptom and seeing a doctor or medical provider or going to the emergency room or being hospitalized for that symptom, at least 1 month after the start of the wildfire. Severe MARI was defined by going to the emergency room or being hospitalized for a respiratory symptom

On average, the wildfire that participants reported as primarily affecting them occurred 8.5 months (SD 2.5) before completion of the survey. The overall prevalence of having any persistent respiratory symptom at the time of the survey was 13.7% (Table 3). The unadjusted prevalence of persistent cough, asthma attacks, bronchitis, and any persistent symptom was higher among participants with medium or high PM_2.5_ exposure, compared to low (Fig. 3). After adjusting for covariates, wildfire-dominated PM_2.5_ exposure during smoky days was associated with significantly increased risk of persistent self-reported asthma attacks (RR 1.28 per SD [1.03–1.59] or RR 1.06 per 10 µg/m^3^ [1.01–1.11]) and suggestion of an increased risk of persistent cough (RR 1.16 per SD [0.99–1.37] or RR 1.03 per 10 µg/m^3^ (1.00–1.07)) and any persistent respiratory symptom (RR 1.13 per SD [0.99–1.3] or RR 1.03 per 10 µg/m^3^ [1.00–1.06]) (Table 3).

### Heterogeneity in risk

**Fig. 4:**
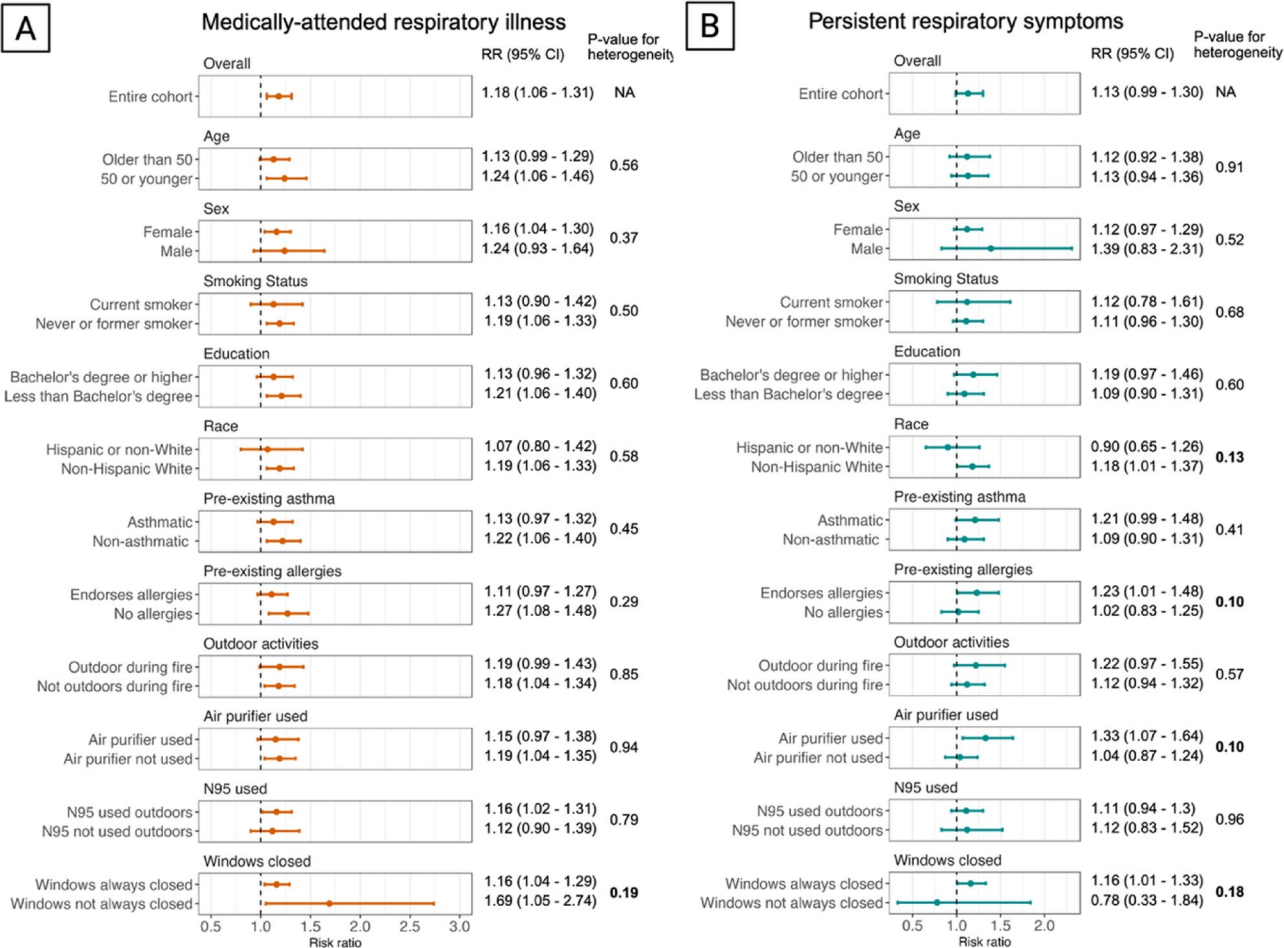
Heterogeneity of the association between wildfire-dominated PM_2.5_ during smoky period and risk of medically-attended respiratory illness (MARI) and any persistent respiratory symptom. Effect modification by covariates that may alter personal wildfire smoke exposure and susceptibility to respiratory disease were explored with the p-value for the interaction term (*p*-value <0.20 bolded) and stratified analyses. **A**: association with MARI. **B**: association with any persistent respiratory symptom. Relative risk is per 1 SD higher wildfire-dominated PM_2.5_ exposure during smoky period. MARI was defined by participant report of respiratory symptom and seeing a doctor or medical provider or going to the emergency room or being hospitalized for that symptom, at least 1 month after the start of the wildfire. Any persistent respiratory symptom was defined as endorsing at least one respiratory symptom at both the time of the survey and at least one other post-wildfire time-period

Exploratory analyses assessed heterogeneity in the association between wildfire-dominated PM_2.5_ and respiratory outcomes across covariates of interest. We examined demographic covariates as well as factors that may affect personal wildfire smoke exposure. The risk of MARI associated with PM_2.5_ exposure was greater among participants who reported not always keeping their windows closed compared to those who reported always keeping their windows closed (Fig. 4). No heterogeneity in risk for MARI was observed for age, sex, education, smoking status, prior asthma, or prior allergies. However, the risk of any persistent respiratory symptom associated with wildfire-dominated PM_2.5_ was higher among participants identifying as non-Hispanic White, and those reporting pre-existing allergies, air purifier use, and keeping windows closed during the wildfire.

The unadjusted prevalence of MARI, severe MARI, any persistent respiratory symptom, persistent asthma attacks, persistent bronchitis, persistent cough, and persistent wheezing following a 2018 wildfire was higher among participants with asthma compared to those without asthma (Table S2). Based on our finding of an association between wildfire-dominated PM_2.5_ and risk of persistent self-reported asthma attacks, we additionally examined whether prior history of asthma modified this risk. We found statistically significant heterogeneity (*p* = 0.02) with RR 1.15 per 10 µg/m^3^ (1.07–1.24) among non-asthmatic participants and RR 1.02 per 10 µg/m^3^ (0.96–1.08) among asthmatic participants. Figure S1 displays the exposure-response curve for probability of persistent asthma attacks by pre-existing asthma.

## Discussion

In this retrospective study of adults without chronic lung disease living in Northern California during a 2018 wildfire event, we report that wildfire-dominated PM_2.5_ exposure is associated with medically-attended respiratory illness (MARI) occurring at least one month after the wildfire and persistent self-reported asthma attacks. Additionally, we found that both intensity and duration of wildfire-dominated PM_2.5_ exposure are associated with future MARI and severe MARI leading to ER visit or hospitalization. These findings serve as evidence that higher PM_2.5_ exposure during a wildfire event can be associated with ongoing respiratory morbidity and consequent healthcare costs beyond the acute wildfire period and may be associated with increased risk of future chronic lung disease.

The potential effects of wildfire smoke exposure on long-term respiratory health in the general population remain poorly understood. It is helpful to use indicators of impaired respiratory health to help us understand the risks to long-term lung health associated with wildfire smoke exposure. We found that one SD higher wildfire-dominated PM_2.5_ exposure during smoky days is associated with 18% increased risk of MARI occurring at least a month after the wildfire. This is consistent with a prior study which found PM_2.5_ concentration during the wildfire season in Montana was associated with influenza rates the following winter [18]. MARI (respiratory exacerbations or acute respiratory illness requiring medical attention) among individuals without chronic lung disease not only lead to missed work [19], impacts on social life [19], and increased health care costs, but have been associated with worse quality of life, greater decline in lung function, risk of progression to chronic obstructive pulmonary disease (COPD), and all-cause mortality [14, 19, 20]. We also found that greater PM_2.5_ exposure during the smoky period is associated with a 28% increased risk of persistent self-reported asthma attacks, and a suggestion of a 16% increased risk of persistent cough which persist for at least 6 months following significant wildfire smoke exposure. Prior studies have shown that persistent wheeze and cough-related symptoms are associated with increased risk of incident obstruction and emphysema [8, 21]. The current study along with prior work which demonstrated greater than expected lung function decline among adults exposed to very high levels of wildfire smoke [22] suggest that wildfire smoke may increase risk of future chronic lung disease. Furthermore, persistent respiratory symptoms among people without lung disease have also been associated with increased risk of future cardiovascular disease events and all-cause death [23, 24]. 

This study also provides insight into potential lasting health effects after a WUI wildfire. The vast majority (> 95%) of participants were exposed to smoke from highly destructive WUI wildfires—the Camp Fire and Carr Fire which were the 1st and 11th most destructive wildfires in California history, respectively. Air pollution, which emanates from burning wood, plastics, insulation, fire-retardant, and heavy metals in WUI wildfires is likely even more toxic than pollution from wildfires which just burn natural vegetation [25, 26]. This is becoming increasingly relevant given that there have been several other devastating WUI wildfires occurring in the U.S. within the last several years, including the 2023 Maui Wildfire and the 2025 Eaton and Palisades fires in Los Angeles. The rise in WUI wildfires is not surprising. The WUI population in the U.S. has grown rapidly (160% rise) in areas with the highest wildfire risk [27]. In conjunction with the increasing size of wildfires due to changes in climate, the growing number of WUI fires has amplified the trend of fires being more deadly and destructive [15]. Our study’s findings of ongoing respiratory symptoms and respiratory illness following exposure to WUI wildfires are consistent with a prior study which examined the health impacts of the 2021 Marshall Fire, a WUI wildfire in Colorado [28]. This study found that 20% of people living within the boundary of the Marshall Fire reported having a dry cough that they believed was related to the wildfire and that had persisted for one year after the fire.

An additional contribution of this study is the finding that both intensity and duration of wildfire smoke exposure are associated with respiratory health beyond the acute wildfire period. These findings suggest that even one day of exposure to high levels of wildfire-dominated PM_2.5_ (peak exposure) may impact risk of future respiratory illness requiring ER visit or hospitalization (severe MARI), and each additional day exposed to PM_2.5_ in the very unhealthy or hazardous range can increase the risk of severe MARI by 11%. We also found that the overall intensity of PM_2.5_ exposure during the wildfire season, as measured by the mean daily PM_2.5_ level during the exposure period, was associated with MARI. Understanding what metrics of wildfire-dominated PM_2.5_ are most strongly associated with risk of ongoing impaired respiratory health can be pivotal for developing strategies to mitigate this risk.

The current study examined the risk of persistent respiratory symptoms and MARI associated with neighborhood-level PM_2.5_ concentration based on participant location. In analyses of heterogeneity, we examined whether participant behaviors that might affect personal smoke exposure may alter the risk of respiratory morbidity associated with outdoor PM_2.5_ level. We observed higher risk of persistent respiratory symptoms associated with wildfire-dominated PM_2.5_ level among people who reported using an air purifier during the wildfire period. This was a surprising finding given that high-efficiency particulate air/arresting (HEPA) filter air purifiers can reduce indoor PM_2.5_ concentrations by 50% or more [29]. Additionally, a small study from 2002 reported that using air purifiers during a wildfire episode was associated with reduced odds of worsening respiratory symptoms after the fire [30]. Unfortunately, our survey did not obtain timing of air purifier use relative to onset of symptoms. It is possible that participants began to use air purifiers after respiratory symptoms appear or after significant smoke exposure has already happened. Therefore, reverse causation may occur whereby the health outcome prompts behavioral changes. Future studies should examine how people are using smoke mitigation techniques when there is a wildfire smoke event (reactively or prophylactically) and which techniques might be effective in reducing long-term morbidity associated with smoke exposure.

We also found that elevated wildfire-dominated PM_2.5_ was associated with greater risk of persistent respiratory symptoms among people with pre-existing allergies compared to those without allergies. There is little data on how wildfire smoke specifically affects allergic disease. One study found that wildfire smoke exposure from the 2018 Camp Fire was associated with increased healthcare use among patients with atopic dermatitis and itch [31]. Additionally, long-term PM_2.5_ exposure has been shown to increase the odds of developing chronic rhinosinusitis [32]. Our study supports further investigation into the impact of wildfire smoke exposure on allergic disease. Interestingly, we found that the although the prevalence of persistent self-reported asthma attacks was higher among participants with pre-existing asthma (13.2% vs. 1.6%), the relative risk associated with wildfire-dominated PM_2.5_ exposure was higher among those without pre-existing asthma. This is likely due to much higher baseline rate of asthma attacks among those with pre-existing asthma. However, it supports that high levels of wildfire smoke exposure is associated with repeated respiratory exacerbations even among those without pre-existing asthma or other chronic lung disease.

Another observation was that non-Hispanic White participants had a greater risk of persistent respiratory symptoms from higher PM_2.5_ exposure than Hispanic and non-White respondents. This finding is contrary to prior studies which have found increased vulnerability to wildfires among Black, Hispanic, and Native American communities [33] and higher risk of respiratory admissions during wildfire smoke waves among Black elderly people compared to White elderly people [34]. In this cohort, 14% identified as Hispanic or non-White which limits robust analyses of specific racial and/or ethnic differences in vulnerability to wildfire smoke exposure. However, the differences seen here may reflect exposure to different wildfires, given geographic variation in the racial and ethnic composition across Northern California counties.

There are several limitations to this study. First, we did not have precise evacuation addresses for many participants who evacuated and therefore relied on imputation of county-level PM_2.5_ estimates for these participants. Second, although we did have data on pre-existing medical conditions such as asthma, allergies and chronic lung disease, we did not have data on pre-existing respiratory symptoms to examine incident respiratory symptoms after the wildfire; however, we do not have reason to believe that this would have been associated with wildfire-dominated PM_2.5_ exposure during the 2018 wildfires, and therefore do not expect this to have significantly confounded the associations we found. Additionally, this study did not isolate wildfire smoke-*specific* PM_2.5_. However, such estimates are inherently uncertain as smoke-specific PM_2.5_ is not measured at large scale and thus cannot be directly validated against ground observations. We therefore used a model to estimate wildfire-dominated PM_2.5_ exposure which has been previously validated and shown to have high correlation with ground PM_2.5_ monitors. Finally, there is risk of recall bias; however, this was mitigated by excluding participants who were affected by 2017 wildfires and had more time between the wildfire and survey completion. Additionally, it is likely that respiratory illness bothersome enough to seek medical attention (MARI and severe MARI) would be less affected by recall bias.

This study has notable strengths including a large sample size, ability to adjust for a wide range of confounders, and robust estimates of individuals’ wildfire-dominated PM_2.5_ exposure which includes evacuation locations. We were also able to examine participants exposed to multiple different wildfires, rather than one single event. Furthermore, the demonstration that respiratory symptoms and MARI persist months after the wildfire smoke has dissipated is a novel contribution to the field which has a paucity of evidence on the intermediate and long-term health consequences of wildfire smoke. This study is also strengthened multiple PM_2.5_ metrics which estimate duration and intensity of wildfire smoke exposure.

## Conclusion

With a dearth of research on wildfires and respiratory health beyond the acute wildfire period, this study takes a longer view. To our knowledge, this investigation provides the strongest evidence to date that exposure to wildfire smoke is associated with medically-attended respiratory illness and persistent self-reported asthma attacks for months after the smoke has cleared. Given that persistent respiratory symptoms and acute respiratory illness are associated with accelerated decline in lung function and future lung function impairment, these results suggest that wildfire smoke exposure may increase risk of future chronic lung disease and therefore may be contributing to respiratory morbidity and mortality beyond the acute wildfire period.

## Supplementary Information

Supplementary Material 1.

## Data Availability

The datasets generated and analyzed during this study are not publicly available due to their identifiable nature (participant address) and privacy concerns. Non-identifiable datasets and code may be requested from the corresponding author.

## Abbreviations

COPD: Chronic obstructive pulmonary disease
EPA: United States Environmental Protection Agency
ER: Emergency room
MARI: Medically-attended respiratory illness
PM_2.5_: Fine particulate matter
RR: Relative risk
SD: Standard deviation
WHAT-Now-CA?: “***W***ildfires and ***H***ealth: ***A***ssessing the ***T***oll in ***No***rthern ***Ca***lifornia Study”
WUI: Wildland-urban interface
