# Supplementary material for: Wildfire smoke exposure and persistent respiratory symptoms and illness: a retrospective cohort study
Source: Respir Res. 2026 May 12;27:275. doi: 10.1186/s12931-026-03694-3 (PMC13340363; doi:10.1186/s12931-026-03694-3)
Supplement: Supplementary file 1 — Supplementary Material 1. [file 12931_2026_3694_MOESM1_ESM.docx]

**Supplementary materials:**

**Expanded methods:**

*Study Design and Population*

This is a retrospective cohort study using data from the ‘***W***ildfires and ***H***ealth: ***A***ssessing the ***T***oll in ***No***rthern ***Ca***lifornia Study’ (WHAT Now, CA) Study. WHAT Now, CA is a longitudinal study which began in January 2018 and enrolled California households after major Northern California wildfires.^21^ In May 2019, an online survey was made available in English and Spanish which focused on participant experiences during and after the 2018 Northern California wildfires. The present analysis included any adult respondent without pre-existing lung disease other than asthma living in Northern California who reported being primarily affected by a wildfire in 2018, and excluded participants who indicated that they were affected by a wildfire that did not occur in 2018 or occurred outside Northern California. Included wildfires were: County Fire (June 2018), Carr Fire (July 2018), Klamathon Fire (July 2018), Mendocino Complex Fire (July 2018), Natchez Fire (July 2018), Ferguson Fire (July 2018), Hirz Fire (August 2018), Donnell Fire (August 2018), Delta Fire (September 2018), Camp Fire (November 2018). The number of participants affected by each fire are presented in **Table S1**. The survey also asked about other household members’ experiences, but this study included only the respondents who took the survey.

**Tables and Figures:**

| **Table S1. Primary wildfires affecting participants in this study** | | |
| --- | --- | --- |
| **Wildfire** | **Smoky period** | **Participants** |
| Camp Fire | 11/8/2018 - 11/20/2018 | 1051 |
| Carr, Delta, Hirz Fires | 7/26/2018 - 9/15/2018 | 265 |
| Mendocino Complex Fire | 7/27/2018 - 9/4/2018 | 37 |
| Klamathon Fire | 7/5/2018 - 8/24/2018 | 11 |
| County Fire | 7/1/2018 - 7/5/2018 | 10 |
| Ferguson Fire | 7/13/2018 - 8/10/2018 | 4 |
| Donnell Fire | 8/1/2018 - 9/4/2018 | 2 |
| Natchez Fire | 7/15/2018 - 8/24/2018 | 1 |
| Total |  | 1381 |
| Wildfires included in this study along with their smoky period and how many participants indicated they were affected by this wildfire. Participants were asked which 2018 wildfire affected them. Smoky periods were determined to start on the first day of the wildfire and end when the mean daily PM_2.5_ concentration for the affected area returned to prior baseline levels. | | |

| **Table S2. Prevalence of respiratory outcomes, stratified by pre-existing self-reported asthma** | | |
| --- | --- | --- |
| **Outcome** | **Prevalence**  **(no asthma)** | **Prevalence**  **(pre-existing asthma)** |
| Any episode of MARI | 181/1056 (17.1%) | 121/325 (37.2%) |
| Any episode of severe MARI | 31/1056 (2.9%) | 25/325 (7.7%) |
| Any persistent respiratory symptom | 112/1056 (10.6%) | 77/325 (23.7%) |
| Persistent asthma attacks | 17/1056 (1.6%) | 43/325 (13.2%) |
| Persistent bronchitis | 14/1056 (1.3%) | 10/325 (3.1%) |
| Persistent cough | 89/1056 (8.4%) | 50/325 (15.4%) |
| Persistent respiratory infections | 14/1056 (1.3%) | 2/325 (0.6%) |
| Persistent wheezing | 37/1056 (3.5%) | 31/325 (9.5%) |
| Unadjusted prevalence of each respiratory outcome, stratified by self-reported pre-existing asthma. MARI was defined by self-report of seeing a doctor or nurse, visiting the ER or being hospitalized for one of the listed respiratory symptoms at least one month after the start of the wildfire. Severe MARI was defined as visiting the ER or being hospitalized for the respiratory symptom. Respiratory symptoms were considered “persistent” if they endorsed having the same symptom “currently,” i.e., at the time the survey was taken (between 6.2 to 19.6 months post fire), and at least one other post-wildfire timepoint (first 3 weeks, 1 month, 3 months, or 6 months after). | | |

| 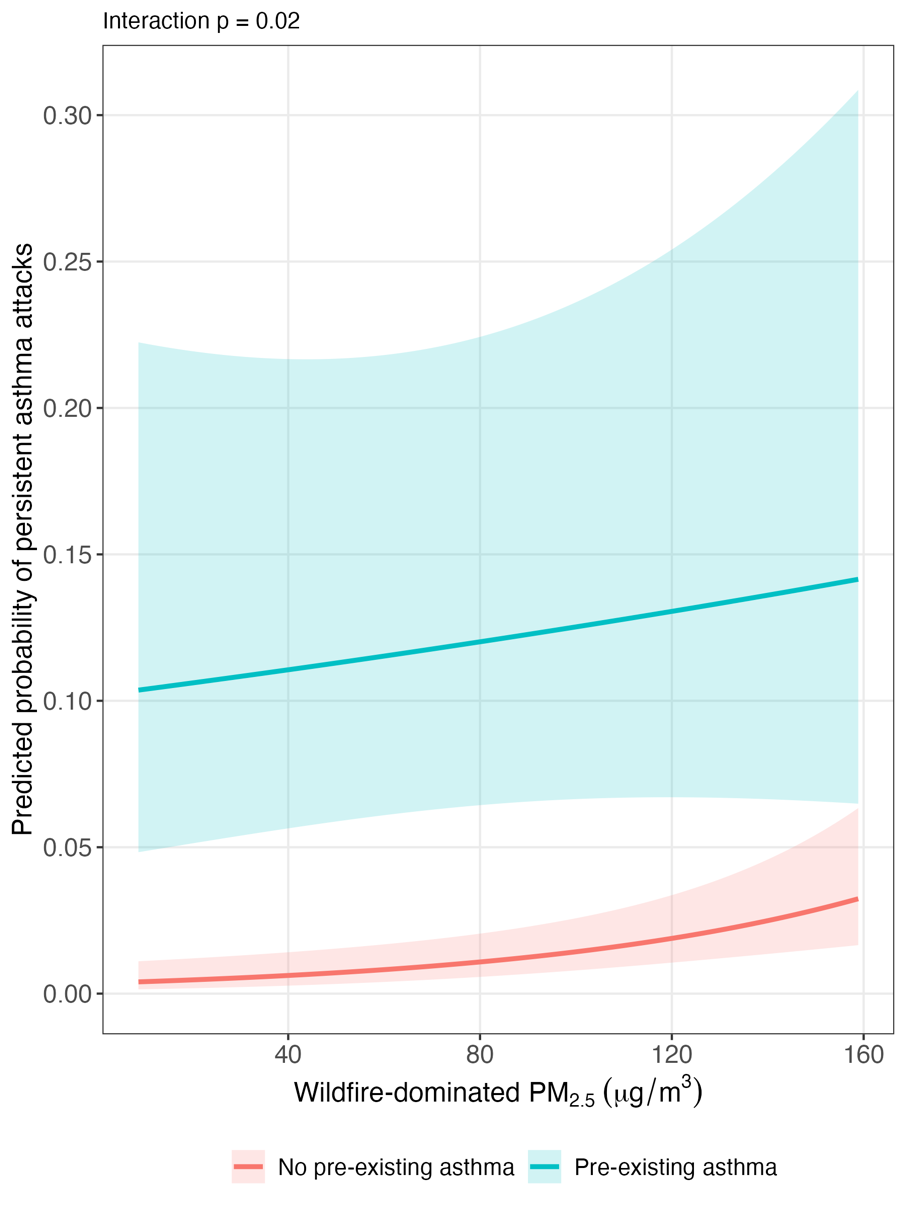 |
| --- |
| **Figure S1. Exposure-response curve for risk of persistent self-reported asthma attacks associated with wildfire-dominated PM_2.5_, stratified by pre-existing asthma.** Predicted probability of persistent asthma attacks estimated using a multivariable Poisson regression model with robust standard error adjusted for age, sex, race/ethnicity (Non-Hispanic White vs all other race/ethnicity groups), current smoking status, pre-existing environmental allergies, education (Bachelor’s degree or higher vs less than Bachelor’s degree). |
